# Visiting Lists

**Published:** 1883-12

**Authors:** 


### VISITING LISTS.

From the publishers of that excellent journal, *The Medical and Surgical Reporter*, of Philadelphia, D. G. Brinton, M. D., editor, we have received The Physicians' Visiting List for 1884, containing more tables than we have here room to enumerate.

We have also to acknowledge the receipt of the visiting list issued by the well known medical publishers, P. Blakiston, Son & Co., full of information useful in every medical emergency. These pocket lists are essential to every physician, and should have a place in the operating case of every dentist, for the proper registering of each prescription and case of office treatment, aside from that inscribed in the dental register.

